# Postpartum quality of life in Indian women after vaginal birth and cesarean section: a pilot study using the EQ-5D-5L descriptive system

**DOI:** 10.1186/s12884-018-2038-0

**Published:** 2018-10-29

**Authors:** Stefan Kohler, Kristi Sidney Annerstedt, Vishal Diwan, Lars Lindholm, Bharat Randive, Kranti Vora, Ayesha De Costa

**Affiliations:** 1 Heidelberg Institute of Global Health, Heidelberg University, Heidelberg, Germany; 2 Division of Global Health, Department of Public Health Sciences, Karolinska Institutet, Stockholm, Sweden; 3 Department of Public Health and Environment, R. D. Gardi Medical College, Ujjain, India; 4 International Centre for Health Research, Ujjain Charitable Trust Hospital and Research Centre, Ujjain, India; 5 Epidemiology and Global Health, Department of Public Health and Clinical Medicine, Umeå University, Umeå, Sweden; 6 Indian Institute of Public Health, Ahmedabad, India

**Keywords:** India, Quality of life, Postpartum period, Vaginal delivery, Episiotomy, Caesarean section, Pilot study

## Abstract

**Background:**

There has been little evaluation of the postpartum quality of life (QOL) of women in India and its association with the mode of birth. This study piloted the use of the generic EQ-5D-5L questionnaire to assess postpartum QOL experienced by rural Indian women.

**Methods:**

A convenience sample of rural women who gave birth in a health facility in Gujarat or Madhya Pradesh was recruited into this pilot study. QOL was measured during three interviews within 30 days of birth using the EQ-5D-5L questionnaire. Patient-level quality-adjusted life days (QALDs) were estimated. Multivariate regression was used to adjust for selected baseline characteristics.

**Results:**

Forty-six women with cesarean section and 178 with vaginal birth from 17 public and private health facilities were studied. Postpartum QOL in both groups improved between interviews 1 and 3. Comparing between vaginal and cesarean births indicated that the vaginal birth group had a higher QOL (0–3 days postpartum: 0.28 vs. 0.57, 3–7 days postpartum: 0.59 vs. 0.81; *P* < 0.001) and was more likely to report no or slight problems in 4 of 5 health dimensions (mobility, self-care, usual activities, pain or discomfort; *P* ≤ 0.04) during interviews 1 and 2. Postpartum QOL converged, but still differed between groups by the time of interview 3 (21–30 days postpartum: 0.85 vs. 0.93; *P* < 0.001). While most women reported no problems by the end of the first postpartum month, the difference in the ability to perform usual activities persisted (*P* = 0.001). In result, fewer QALDs were attained by women in the cesarean section group between day 1 and day 21 postpartum (13.1 vs. 16.6 QALDs; *P* < 0.001). Subgroup analysis showed that having had an episiotomy during vaginal birth was also associated with reduced QOL postpartum, but to a lesser extent than cesarean section. Similar results were obtained when adjusting for socioeconomic, pregnancy and birth characteristics, but postpartum QOL already ceased to be statistically different between groups before interview 3.

**Conclusions:**

Vaginal births, even with episiotomy, were associated with a higher postpartum QOL than cesarean births among the Indian women in our pilot study. Finding these expected results suggests that the EQ-5D-5L questionnaire is a suitable instrument to assess postpartum QOL in Indian women.

## Background

The postpartum period is sometimes referred to as the fourth stage of labor. It begins after having given birth and may extend up to six months after giving birth [1]. Returning to the prepregnant state, women who gave birth experience physiological and psychological changes and continue to have increased health risks, particularly during the first weeks of the postpartum period. Postpartum health risks include physical health risks, like anemia, infections or wound healing complications, as well as mental health risks, like anxiety, depression, fatigue or stress [1, 2], which can bring about various degrees of morbidity in the postpartum period.

The number of women experiencing pregnancy related morbidity in the postpartum period is estimated to be magnitudes greater than the number who die [3]. Yet, there is little reliable information on the prevalence of maternal morbidity and postpartum quality of life (QOL), specially from low income settings [4–6].

In India, despite the implementation of large programs focused on intrapartum care, an emphasis on the provision of postpartum care and its evaluation is lacking. The latter has partly been due the absence of appropriate measuring instruments until quite recently, when the Mother Generated Index (MGI) and the Maternal Postpartum Quality of Life (MAPP-QOL) questionnaires have become available [7, 8]. However, the MGI is qualitative in nature and requires an educated trained health worker to administer the questionnaire. The MAPP-QOL, in turn, is a self-administered, paper and pencil questionnaire that requires women to be able to read and write. These characteristics limit the usability of the existing, specific postpartum QOL questionnaires in large-scale studies and among women with low levels of literacy.

In the absence of validated simple-to-implement instruments for measuring the QOL of pregnant or postpartum women, multiple studies have assessed the QOL of pregnant and postpartum women using generic instruments. Most of these studies were conducted in high income countries [4].

We used the generic EQ-5D questionnaire with five levels (EQ-5D-5L) for assessing the postpartum QOL experienced by rural Indian women in a pilot study. Our aims were to determine the feasibility of applying the EQ-5D-5L questionnaire to postpartum women in rural India and to explore whether the instrument can detect differences in postpartum QOL among rural Indian women who delivered vaginally or by caesarean section. Specific objectives were, first, to measure the QOL of Indian women in the first month postpartum; second, to examine which dimensions of QOL have been affected and, third, to assess postpartum health based on quality-adjusted life days (QALDs).

## Methods

### Study design and setting

This was a prospective pilot study of the QOL of postpartum women living in rural areas of the Indian states of Gujarat and Madhya Pradesh. Postpartum women were recruited in the two purposively selected districts of Ujjain in Madhya Pradesh and Surendranagar in Gujarat. Only women who lived within these district were included into the study to facilitate follow-ups in their community after discharge from hospital.

Women from Ujjain district in Madhya Pradesh who gave birth in April 2016 were recruited either in the government district hospital or the non-governmental teaching medical hospital of the district. In Gujarat, women who gave birth between July and November 2014 were recruited in a community health center and 14 private hospitals of Surendranagar district. Women were purposively selected to include vaginal births and cesarean sections in the sample. Vaginal births included vaginal births with episiotomies, but no forceps or vacuum assisted births. Cesarean sections included both emergency and elective procedures.

Women were approached three times after giving birth. Visits for an interview occurred 0–3 days, 3–7 days, and 21–30 days postpartum. The first administration of the EQ-5D-5L questionnaire was in the health facility. During the first interview socioeconomic, pregnancy and birth characteristics of the women were elicited. Follow-ups were conducted in women’s homes for discharged women, or in the health facilities for a minority of women who were still in the health facility at the scheduled follow-up times. At all visits, women were interviewed by the same research assistant.

The study was part of the MATIND research projected, which evaluated two large scale demand side financing programs for maternal health in India – the Chiranjeevi Yojana (CY) scheme and the Janani Suraksha Yojana (JSY) scheme [9]. Early stage MATIND research demonstrated that many women accessing the CY and JSY schemes lack formal education and are part of traditional Indian societies [10, 11], in which women often have low levels of autonomy and freedom of expression.

### Quality of life measurement

Health-related QOL was assessed based on women’s responses to the EQ-5D-5L questionnaire. The EQ-5D is a two-part instrument. Part one, the so-called descriptive system, is a self-assessment of today’s health along 5 pre-specified dimensions: mobility, self-care, usual activity, pain or discomfort, and anxiety or depression. For each dimension, the respondent selects one of 5 levels of incapacitation in the 5L version: no, slight, moderate, severe, or extreme problems. The combination of answers can result in 5^5^ = 3125 possible health states. Part two of the EQ-5D consists of a visual analogue scale (VAS) which can be used to obtain a visually guided self-rating of today’s health on a scale from 0 (worst) to 100 (best). Both parts of the EQ-5D instrument should be used together [12], but women in the pilot study women had difficulties to assess their health using a VAS. Thus, use of the EQ VAS was ceased within the pilot study.

### Data collection and analysis

We used the Hindi version of EQ-5D-5L health questionnaire with permission from the EuroQoL Group [13, 14]. The questionnaire was intended to be administered to each recruited woman by a trained research assistant at three points in time: 1, 7 and 21 days after birth or as close as possible to these dates. In practice, the first interview occurred between 0 and 3 days postpartum, the second interview between 3 and 7 days postpartum, and the final interview between 21 and 30 after women gave birth. Women’s characteristics were obtained at baseline from hospital records and staff as well as by interviewing women. Interviews took place in a language understood by both the woman and the research assistant. No sample size calculation was made for this pilot study.

Problem levels, reported by the postpartum women through answering the descriptive system of the EQ-5D-5L at three different points in time, were mapped to QOL weights based on a value set obtained from the general population in the United Kingdom by time trade-off valuation techniques. We used the United Kingdom value set due to the lack of an EQ-5D population norm for India [15]. Furthermore, we used a crosswalk value set that allows to link responses to the EQ-5D-5L with the existing value sets for the EQ-5D-3L [16], as only value sets for the 3-level version of the EQ-5D were available at the time of analysis.

Quality-adjusted life time was calculated based on days (QALDs) rather than years (QALYs) due to the brief postpartum period studied. QALDs were estimated on the individual-level by applying the area-under-the-curve method [17, 18]. The area-under-the-curve method was implemented summing up, by birth mode and episiotomy status, the areas of under the curves obtained from linearly connecting each woman’s QOL values from day 1 to day 21 postpartum.

To obtain comparable QALDs, despite some variation in the postpartum days when QOL data was collected, we standardized the reference period for which QALDs are reported to days 1 to 21 postpartum. For women surveyed in Gujarat, we used recorded interview dates to impute QOL weights for days 1 and 21 postpartum based on linear interpolation. In one instance, interpolation led to a QOL weight greater than one and was set to one, the weight for the best health status. For women surveyed Madhya Pradesh, we assumed that all interviews took place exactly 1, 7 and 21 days after birth.

In the descriptive analysis, differences in continuous variables were assessed using Wilcoxon rank-sum and two-sample *t*-tests. Differences in proportions were compared using Pearson’s chi-squared tests. Hypotheses about regression coefficients were assessed based Wald tests. Generalized estimating equation was used to estimate the adjusted association between time and QOL, assuming a Gaussian distribution of QOL, an identity link function and unstructured correlation between the data obtained over three interview visits. Linear regression was used to examine the association between birth mode and QALDs, controlling for characteristics that may affect postpartum QOL.

## Results

### Participant characteristics

In total, 231 women were recruited into the pilot study: 60 in Ujjain district of Madhya Pradesh and 171 in Surendranagar district of Gujarat. Seven women were excluded from the analysis: one woman who gave birth on the way to a hospital, two women who experienced a still birth or neonatal death, and four women who could not be contacted for follow-up interviews.

The final sample included 224 women, of which 178 (79%) gave vaginal birth and 46 (21%) gave birth by cesarean section. Socioeconomic, pregnancy and birth characteristics of these women are shown in Table 1. Women were aged 18 to 36 years. Those who had a vaginal birth were more likely to belong to a socially and educationally disadvantaged group and a poorer household. The state specific Indian poverty line considers several other factors than income, and similar portions of women in both birth groups were below poverty line.

The number of previous pregnancies ranged from 0 to 7 in both birth groups with the same median of one previous pregnancy. An episiotomy was reported by 67% of the women who gave vaginal birth and by one woman with a cesarean section. In the vaginal birth group, 10% of women experienced one or more of the following complications during their birth or pregnancy: premature rupture of membranes, preeclampsia signs, obstructed labor, malpresentation, oligohydramnios, previous cesarean section. By contrast, 87% of the women with a cesarean section had at least one of these complications that increase the likelihood of cesarean section. No women in the sample was diagnosed with eclampsia or had convulsions. Similar portions of 87% and 91% of the newborns were healthy in both groups 2–3 days postpartum. Cesarean sections were more likely to happen in a private hospital, associated with longer hospital stays, and all performed by a medical doctor.

**Table 1:** Socioeconomic, pregnancy and birth characteristics of women in the pilot study by birth mode

	Vaginal birth	Cesarean section	*P*
*Socioeconomic characteristics*			
Mother’s age			0.49
18–20	39 (21.9%)	6 (13.0%)	
21–24	84 (47.2%)	23 (50.0%)	
25–29	46 (25.8%)	13 (28.3%)	
30–36	9 (5.1%)	4 (8.7%)	
Years of schooling			0.007
None	53 (29.8%)	8 (17.4%)	
Primary (1–5)	35 (19.7%)	4 (8.7%)	
Secondary (6–12)	87 (48.9%)	30 (65.2%)	
Higher (> 12)	3 (1.7%)	4 (8.7%)	
Caste of woman			< 0.01
Scheduled Caste/Tribe	45 (25.3%)	7 (15.2%)	
Other Backward Caste	125 (70.2%)	31 (67.4%)	
General Caste	8 (4.5%)	8 (17.4%)	
Household income			0.01
0–2499 INR	16 (9.0%)	1 (2.2%)	
2500–4999 INR	49 (27.5%)	5 (10.9%)	
5000–9999 INR	68 (38.2%)	20 (43.5%)	
10,000–60,000 INR	45 (25.3%)	20 (43.5%)	
Below poverty line			0.84
No	40 (22.5%)	11 (23.9%)	
Yes	138 (77.5%)	35 (76.1%)	
*Pregnancy and birth characteristics*			
Previous pregnancies			0.42
None	23 (12.9%)	10 (21.7%)	
1–2	125 (70.2%)	31 (67.4%)	
3–4	24 (13.5%)	4 (8.7%)	
5−7	6 (3.4%)	1 (2.2%)	
Woman reported episiotomy			< 0.001
No	58 (32.6%)	45 (97.8%)	
Yes	120 (67.4%)	1 (2.2%)	
Risk factors for cesarean section^1^			< 0.001
No	160 (89.9%)	6 (13.0%)	
Yes	18 (10.1%)	40 (87.0%)	
Ante- or postpartum hemorrhage			0.40
No	169 (94.9%)	45 (97.8%)	
Yes	9 (5.1%)	1 (2.2%)	
Prolonged labor, anemia or fever			0.72
No	151 (84.8%)	40 (87.0%)	
Yes	27 (15.2%)	6 (13.0%)	
Baby’s health (2–3 days postpartum)			0.43
Healthy	155 (87.1%)	42 (91.3%)	
Sick	23 (12.9%)	4 (8.7%)	
Length of hospital stay after birth, median (IQR)	2.0 (1.0, 2.0)	3.0 (3.0, 5.0)	< 0.001
Length of hospital stay			< 0.001
Below median duration for birth mode	81 (45.5%)	7 (15.2%)	
Of median duration for birth mode	81 (45.5%)	23 (50.0%)	
Above median duration for birth mode	16 (9.0%)	16 (34.8%)	
Birth place			0.02
Community health center	55 (30.9%)	6 (13.0%)	
District hospital	39 (21.9%)	8 (17.4%)	
Private hospital	84 (47.2%)	32 (69.6%)	
N	178	46	

^1^Risk factors for cesarean section include premature rupture of membranes, preeclampsia signs, obstructed labor, malpresentation, oligohydramnios, and previous cesarean section

*INR* Indian Rupee, *IQR* interquartile range

*P*-values of Wilcoxon rank-sum test or Pearson’s chi-squared test

### Feasibility of using the EQ-5D

The pilot study started administering both parts of the EQ-5D-5L questionnaire. We observed early in the study that participating women had difficulties with the VAS component of the EQ-5D questionnaire. Given the standard instructions, several respondents were uncertain of where to place a mark on the VAS. Therefore, a decision was made to pursue the pilot study further using only the descriptive system of the EQ-5D-5L questionnaire through which we obtained the results presented below.

### Postpartum quality of life

#### Descriptive analysis

**Fig. 1:**
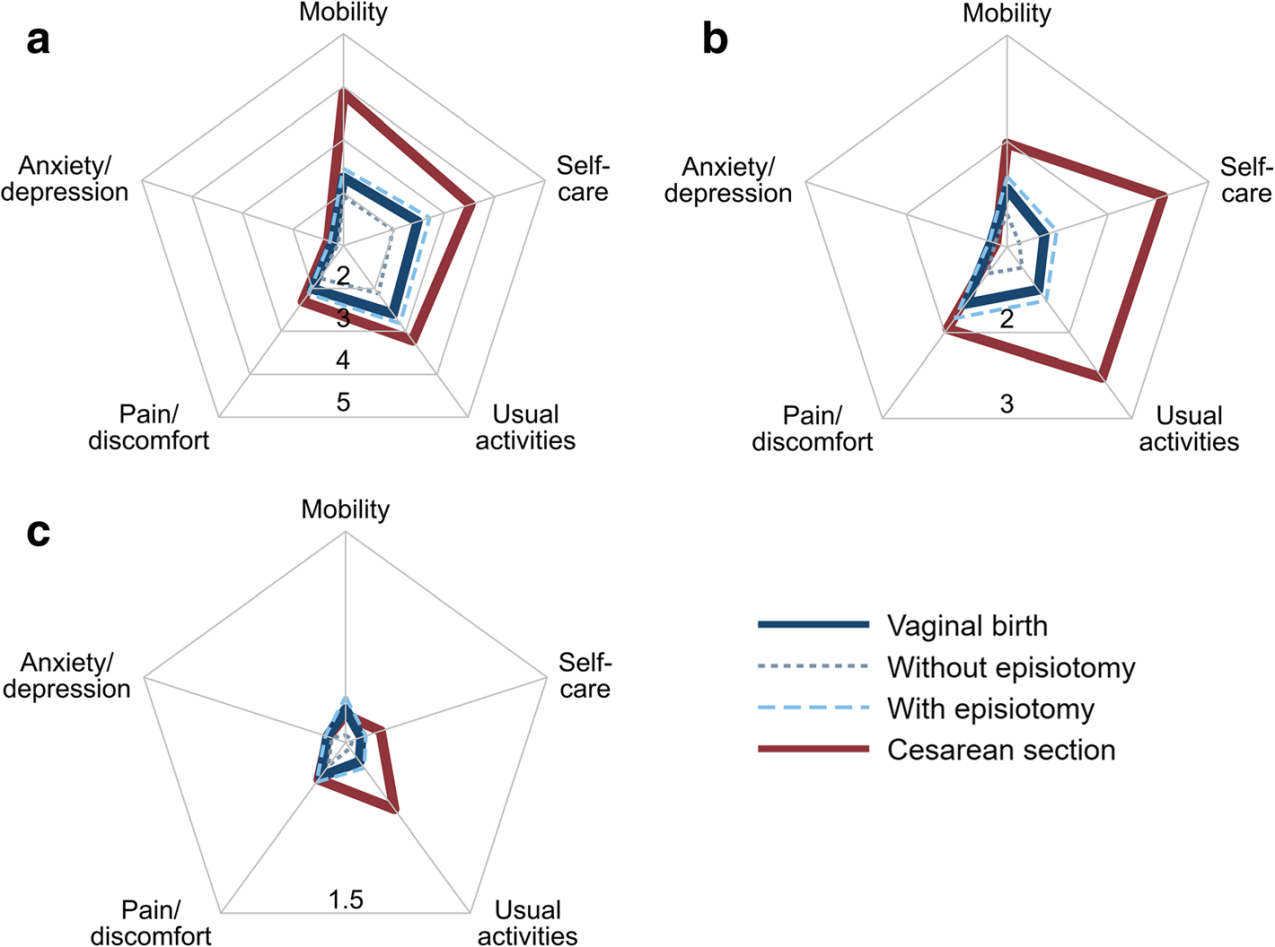
Mean level of postpartum problems in the EQ-5D health dimensions by birth mode and time passed since giving birth. (**a**) 0–3 days postpartum, (**b**) 3–7 days postpartum, (**c**) 21–30 days postpartum. *N* = 224. In the EQ-5D-5L questionnaire, postpartum women rated each of five health dimensions (mobility, self-care, usual activities, pain/discomfort and anxiety/depression) as associated with no problems = 1, slight problems = 2, moderate problems = 3, severe problems = 4, unable to/extreme  problems = 5 on the day of the interview. Higher problem levels result in lower postpartum quality of life

Postpartum women rated each of five health dimensions (mobility, self-care, usual activities, pain or discomfort, and anxiety or depression) in the EQ-5D-5L questionnaire as associated with no problems = 1, slight problems = 2, moderate problems = 3, severe problems = 4, unable to/extreme problems = 5. Assuming the numbers assigned to these problem levels are such that numerically equal differences in ratings represent equal differences in the levels of problems, Fig. 1 shows the mean levels of incapacitation for each dimension, by birth mode and the time passed since giving birth. On average, women with cesarean section perceived stronger problems than women with vaginal birth in 4 out of 5 health dimensions assessed by the EQ-5D. The level of reported problems with anxiety or depression was low across birth modes. Receiving an episiotomy during vaginal birth reduced postpartum QOL in the same health dimensions as having a cesarean section, but, overall, to a lesser extent. For all birth modes, reported problems decreased in the observed postpartum period.

The percentages of women reporting problem levels 1 to 5 for each of the five dimensions of the EQ-5D-5L are shown in Table 2. Within the first three days of birth, the portions of women reporting no problems were substantially higher, in both birth groups, for anxiety or depression (≥74%) than for the other four dimensions (self-care, mobility, usual activities, and pain or discomfort; <47%, *P* < 0.001) The respective severity of problems varied significantly with the birth mode in all of the latter four dimensions up to 3–7 days after birth (*P* ≤ 0.04). By the time of the last interview 21–30 days postpartum, ≥ 89% of women, regardless of the mode of their recent birth, reported no problems with mobility, self-care, pain or discomfort, and anxiety or depression, regardless of the mode of their recent birth. No women indicated extreme problems during the last interview, ≤0.6% severe problems, ≤2.2% moderate problems, and <20% slight problems in any of the EQ-5D health dimensions.

**Table 2:** Comparison of problems by EQ-5D health dimension in women with vaginal birth and cesarean section within the first month postpartum

Health dimension	Problem level	0–3 days postpartum			3–7 days postpartum			21–30 days postpartum		
		VB (%)	CS (%)	*P*	VB (%)	CS (%)	*P*	VB (%)	CS (%)	*P*
Mobility	No problems	24.7	4.3	< 0.001	53.4	39.1	0.004	93.3	93.5	0.75
	Slight problems	48.3	17.4		39.3	39.1		5.6	6.5	
	Moderate problems	11.2	4.3		6.2	13.0		1.1		
	Severe problems	5.1	34.8		1.1	2.2				
	Unable to	10.7	39.1		0.0	6.5				
Self-care	No problems	23.0	2.2	< 0.001	69.7	23.9	< 0.001	96.1	91.3	0.18
	Slight problems	52.2	32.6		27.0	50.0		3.9	8.7	
	Moderate problems	1.7	17.4		1.7	0.0				
	Severe problems	0.6	6.5		0.0	0.0				
	Unable to	22.5	41.3		1.7	26.1				
Usual activities	No problems	20.8	19.6	0.01	60.1	28.3	< 0.001	95.5	80.4	0.001
	Slight problems	46.6	23.9		35.4	39.1		3.9	19.6	
	Moderate problems	8.4	10.9		1.1	8.7				
	Severe problems	1.1	6.5		1.1			0.6		
	Unable to	23.0	39.1		2.2	23.9				
Pain or discomfort	No	27.0	28.3	0.008	47.8	28.3	0.04	92.1	89.1	0.28
	Slight	48.9	30.4		39.3	47.8		5.6	10.9	
	Moderate	21.3	26.1		11.8	23.9		2.2		
	Severe	2.2	13.0		1.1					
	Extreme	0.6	2.2							
Anxiety or depression	No	82.6	73.9	0.51	88.2	91.3	0.83	96.6	97.8	0.51
	Slight	15.2	23.9		9.0	6.5		2.2		
	Moderate	0.6			1.7	2.2		1.1	2.2	
	Severe	1.7	2.2		1.1					
	Extreme									
N		178	46		178	46		178	46	

*VB* vaginal birth, *CS* cesarean section

Only the ability to perform usual activities continued to be significantly different between women with vaginal and cesarean births towards the end of the first postpartum month (96 vs. 80% without problems; *P* = 0.001). Anxiety or depression was the only dimension for which the data did not indicate a difference by birth mode throughout the whole first month postpartum (*P* ≥ 0.51). The highest shares of extreme problems for the dimensions of mobility, self-care, usual activities, and pain or discomfort as well as most severe problems occurred in the cesarean section group shortly after birth.

**Fig. 2:**
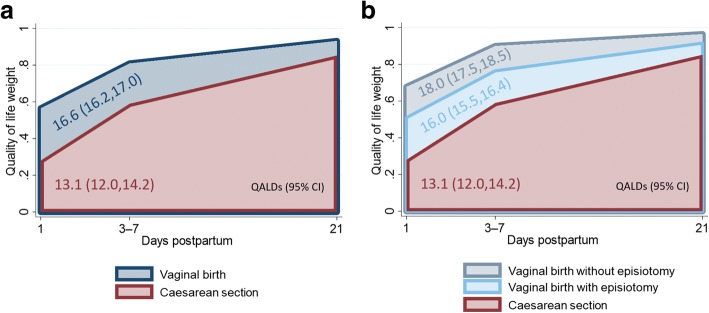
Indian women’s quality of life days (QALDs) in the first 21 days postpartum by birth mode. (**a**) All types of vaginal births vs. cesarean sections. (**b**) Vaginal births without episiotomy vs. vaginal births with episiotomy vs. cesarean sections. N = 224. Graphs are a simplification due drawing the mean of all QOL weights obtained 3–7 days postpartum at 7 days. QOL weights at postpartum days 1 and 21 were obtained by linear interpolation of the two QOL weights observed closest to the respective day

For an overall assessment of the health problems reported in the five dimensions assessed by the EQ-5D, the reported problem levels were mapped into to QOL weights. Table 3 compares the unadjusted QOL weights between vaginal and cesarean births. The QOL weights quantify how postpartum QOL improved between interviews 1 and 3 in both groups, and they indicate that the vaginal birth group had a higher QOL throughout in comparison to women after cesarean section. In result, fewer QALDs were attained by women in the cesarean section group between day 1 and day 21 postpartum (13.1 vs. 16.6 QALDs, *P* < 0.001; see Fig. 2a). Subgroup analysis showed that having had an episiotomy during vaginal birth was also associated with reduced postpartum QOL in comparison to a vaginal birth without episiotomy (16.0 vs. 18.0 QALDs; *P* < 0.001), but to a lesser extent than giving birth by cesarean section (13.1 vs. 18.0 QALDs, *P* < 0.001; see Fig. 2b).

**Table 3:** Comparison of quality of life weights and quality-adjusted life days in women with vaginal birth and cesarean section within the first month postpartum

	Vaginal birth			Cesarean section
	All	No episiotomy	Episiotomy	
	Mean (95% CI)	Mean (95% CI)	Mean (95% CI)	Mean (95% CI)
QOL weight				
0–3 days postpartum	0.57 (0.52, 0.61)	0.68 (0.6, 0.75)	0.51 (0.46, 0.56)	0.28 (0.18, 0.38)
3–7 days postpartum	0.81 (0.78, 0.84)	0.9 (0.87, 0.93)^a^	0.76 (0.73, 0.8)	0.59 (0.51, 0.67)
21–30 days postpartum	0.93 (0.92, 0.94)	0.96 (0.95, 0.98)^a^	0.92 (0.9, 0.93)	0.85 (0.82, 0.89)
QALDs (1–21 days postpartum)	16.6 (16.2, 17.0)	18.0 (17.5, 18.5)	16.0 (15.5, 16.4)	13.1 (12.0, 14.2)
N	178	58	120	46

*QALDs* quality-adjusted life days, *QOL* quality of life

Two-sample *t*-tests and Wilcoxon rank-sum tests indicate significant differences in the means and distributions of the QOL weights and QALDs in the following comparisons: all vaginal births vs. cesarean section, no episiotomy vs. episiotomy, no episiotomy vs. cesarean section, and episiotomy vs cesarean section; *P* < 0.001. QOLs weights increased significantly with the time passed since birth in each of the four birth groups distinguished; *P* < 0.001 for all comparisons between visits, except for the following comparison: ^a^ *P* = 0.04

#### Multivariate analysis

Results similar to those of the descriptive analysis were obtained when adjusting for selected baseline characteristics. Table 4 presents generalized estimating equation models 2 of the QOL weight at the postpartum interview times and linear regression models 2 of the QALDs in the first 21 postpartum days. Models (a) compare women who gave vaginal birth and women who gave birth by cesarean section. Models (b) further distinguish whether an episiotomy was performed during vaginal birth. All regression models adjusted for the socioeconomic, pregnancy and birth characteristics reported in Table 1.

**Table 4:** Adjusted comparison of quality of life weights and quality-adjusted life days in women with vaginal birth and cesarean section within the first month postpartum

	QOL weight		QALDs (1–21 days postpartum)	
	Model 1a	Model 1b	Model 2a	Model 2b
	Coef. (95% CI)	Coef. (95% CI)	Coef. (95% CI)	Coef. (95% CI)
Vaginal birth (all)			14.6 (12.3, 16.9)^m^	
0–3 days postpartum	0.39 (0.27, 0.52)^a^			
3–7 days postpartum	0.64 (0.52, 0.76)^b^			
21–30 days postpartum	0.76 (0.65, 0.88)^c^			
Vaginal birth (no episiotomy)				17.1 (14.6, 19.6)^n,o^
0–3 days postpartum		0.52 (0.35, 0.7)^d,e^		
3–7 days postpartum		0.75 (0.61, 0.89)^f,g^		
21–30 days postpartum		0.81 (0.67, 0.95)^h,i^		
Vaginal birth (episiotomy)				15.3 (131, 17.6)^n,p^
0–3 days postpartum		0.41 (0.27, 0.54)^d,j^		
3–7 days postpartum		0.66 (0.53, 0.79)^f,k^		
21–30 days postpartum		0.81 (0.69, 0.94)^h,l^		
Cesarean section			11.3 (8.94, 13.7)^m^	12.5 (10.1, 14.8)^o,p^
0–3 days postpartum	0.15 (0.01, 0.29)^a^	0.2 (0.05, 0.35)^e,j^		
3–7 days postpartum	0.46 (0.33, 0.59)^b^	0.51 (0.37, 0.65)^g,k^		
21–30 days postpartum	0.73 (0.62, 0.83)^c^	0.77 (0.65, 0.89)^i,l^		
Mother’s age (ref. = 21–24)				
18–20	0.03 (−0.02, 0.08)	0.03 (−0.02, 0.08)	0.49 (−0.49, 1.48)	0.36 (−0.61, 1.33)
25–30	−0.01 (−0.07, 0.04)	−0.02 (−0.07, 0.03)	−0.52 (−1.6, 0.56)	−0.6 (−1.64, 0.45)
30–36	0.04 (−0.03, 0.11)	0.03 (−0.04, 0.11)	0.89 (−0.68, 2.45)	0.64 (−0.96, 2.24)
Years of schooling (ref. = secondary: 6–12)				
None	0.06 (0.01, 0.11)	0.06 (0.01, 0.1)	0.33 (−0.66, 1.32)	0.3 (−0.65, 1.25)
Primary (1–5)	0.06 (0.01, 0.12)	0.06 (0.01, 0.11)	0.756 (−0.332, 1.83)	0.69 (−0.36, 1.75)
Higher (> 12)	−0.13 (−0.23, −0.02)	−0.13 (−0.23, −0.02)	−2.22 (−4.44, −0.002)	−2.18 (−4.53, 0.17)
Caste of woman (ref. = Other Backward Caste)				
Scheduled Caste/Tribe	0.02 (−0.02, 0.06)	0.02 (−0.02, 0.06)	−0.17 (−1.06, 0.72)	−0.14 (−0.99, 0.71)
General Caste	0.14 (0.04, 0.23)	0.13 (0.04, 0.22)	1.75 (−0.23, 3.74)	1.64 (−0.27, 3.54)
Household income (ref. = 2500–4999 INR)				
0–2499 INR	0.1 (0.03, 0.18)	0.09 (0.02, 0.17)	1.13 (−0.19, 2.44)	0.85 (−0.37, 2.08)
5000–9999 INR	0.06 (0.01, 0.12)	0.05 (0.001, 0.11)	0.66 (−0.35, 1.68)	0.48 (−0.52, 1.47)
10,000–60,000 INR	0.03 (−0.01, 0.08)	0.03 (−0.01, 0.08)	0.08 (−0.92, 1.07)	0.09 (−0.88, 1.07)
Above poverty line	0.04 (−0.01, 0.09)	0.03 (−0.02, 0.08)	0.8 (−0.24, 1.85)	0.6 (−0.42, 1.61)
Previous pregnancies (ref. = 1–2)				
None	0.08 (−0.01, 0.16)	0.04 (−0.05, 0.13)	1.11 (−0.39, 2.61)	0.25 (−1.22, 1.73)
3–4	0.14 (0.04, 0.25)	0.08 (−0.03, 0.2)	2.05 (0.15, 3.96)	0.49 (−1.51, 2.49)
5–7	0.05 (−0.08, 0.17)	−0.02 (−0.16, 0.12)	1.27 (−1.31, 3.85)	−0.42 (−3.06, 2.22)
Risk factors for cesarean section^1^	−0.003 (−0.06, 0.06)	−0.00108 (−0.06, 0.06)	0.36 (−0.85, 1.56)	0.4 (−0.76, 1.56)
Ante- or postpartum hemorrhage	−0.03 (−0.12, 0.07)	− 0.02 (−0.11, 0.07)	−0.69 (−2.39, 1.02)	−0.52 (−2.07, 1.02)
Prolonged labor, anemia or fever	0.03 (−0.02, 0.08)	0.03 (−0.02, 0.08)	0.33 (−0.56, 1.22)	0.35 (−0.53,1.23)
Baby is sick (2–3 days postpartum)	−0.01 (−0.04, 0.01)	−0.02 (−0.05, 0.01)	−0.27 (−0.89, 0.34)	−0.39 (−1.01, 0.23)
Length of hospital stay (ref. = median duration for birth mode)				
< Median duration for birth mode	−0.03 (−0.09, 0.02)	−0.03 (−0.08, 0.03)	0.33 (−1.02, 1.69)	0.45 (−0.87, 1.77)
> Median duration for birth mode	−0.04 (−0.11, 0.04)	−0.04 (−0.11, 0.03)	0.04 (−1.45, 1.54)	−0.06 (−1.47, 1.35)
Health facility (ref. = private hospital)				
Community health center	0.05 (−0.02, 0.11)	0.05 (−0.02, 0.11)	0.29 (−1.24, 1.82)	0.37 (−1.12, 1.87)
District hospital	0.12 (0.04, 0.21)	0.09 (0.003, 0.18)	1.09 (−0.41, 2.59)	0.24 (−1.33, 1.82)
R^2^			0.97	0.97
N	224 × 3	224 × 3	224	224

^1^Risk factors for cesarean section include premature rupture of membranes, preeclampsia signs, obstructed labor, malpresentation, oligohydramnios, and previous cesarean section

^a^ *P* < 0.001, ^b^ *P* = < 0.001, ^c^ *P* = 0.30, ^d^ *P* = 0.01, ^e^ *P* < 0.001, ^f^ *P* = 0.008, ^g^ *P* < 0.001, ^h^ *P* = 0.91, ^i^ *P* = 0.28, ^j^ *P* < 0.001, ^k^ *P* = 0.002, ^l^ *P* = 0.20, ^m^ *P* < 0.001, ^n^ *P* < 0.001, ^o^ *P* < 0.001, ^p^ *P* < 0.001. QOL weights increased significantly with the time passed since birth in each of the four birth groups distinguished; *P* < 0.001 for all comparisons between interview visits

*QALDs* quality-adjusted life days, *QOL* quality of life. *Models 1* generalized estimating equation of QOL weights, *models 2* linear regression of QALDs. Sampling weights were used to resemble a population with an equal number of normal vaginal deliveries and cesarean sections. R^2^ from linear regression models without intercept

In models 1, the lowest QOL weights were estimated immediately after birth, irrespective of the birth mode and whether an episiotomy was performed or not. On average, QOL improved for all women between interviews 1 and 3 as indicated by significantly increasing QOL coefficients from 0–3 days to 21–30 days postpartum for both birth modes. Women with a vaginal birth, for instance, had an average QOL weight of 0.40 at the time of interview 1, which significantly increased to 0.64 at the time of interview 2 and to 0.76 at the time of interview 3 (*P* < 0.001). An episiotomy significantly lowered the estimated QOL weights after vaginal birth in comparison to a vaginal birth with no episiotomy until the time of interview 2 (3–7 days postpartum; *P* ≤ 0.008) but not afterwards.

How the estimated differences in women's postpartum QOL weights accumulate over time is captured by the QALDs outcome in models 2. On average, women had more QALD during the first 21 postpartum days after vaginal birth than after cesarean section (14.6 vs. 11.3 QALDs; *P* < 0.001). A vaginal birth with an episiotomy was associated with significantly fewer QALDs in the first 21 postpartum days than a vaginal birth without episiotomy (15.3 vs. 17.1 QALDs; *P* < 0.001), but with significantly more QALDs in the first 21 postpartum days than a cesarean section (15.3 vs. 12.5 QALDs; *P* < 0.001).

## Discussion

### Motivation for piloting the EQ-5D questionnaire with rural Indian postpartum women

As health care becomes more patient-centered, patient-reported outcomes such as QOL are becoming increasingly important [19, 20]. The few studies that assessed postpartum QOL in India prior to our study have used the MGI questionnaire [21, 22]. Related to a lack of research on postpartum QOL in Indian women [23], there is limited knowledge and experience on choice of a suitable questionnaire for the study postpartum QOL in India.

We tested using the generic EQ-5D questionnaire for postpartum QOL assessment in India by interviewing a convenience sample of rural women three times with the EQ-5D-5L descriptive system in their first month postpartum. The rationale behind testing the EQ-5D questionnaire for postpartum QOL assessment in rural Indian women was that existing specific instruments for postpartum QOL, like the MGI or MAPP-QOL questionnaire, can be impractical for large-scale use in low-literacy populations and in settings in which women have low levels of autonomy and freedom of expression.

The MGI measures subjective QOL and, unlike the EQ-5D, does not consist of a predefined checklist of problems. Specifically, the MGI consist of three steps during which a woman generates her own QOL index. Step 1 requires a woman to identify up to eight most important areas of her life that have been affected by having a baby, and to indicate if she thinks these areas are positive, negative, both or neither of these. Step 2 asks a woman to score, on a visual analogue scale from 0 to 10, how she has been affected in each identified area over the past month. Step 3 requires a woman to allocate 20 points across the identified areas, according to how important an area is to her QOL. Thus, applying the MGI requires settings in which women are able, have time and are given time to think about and articulate such issues. In studies including larger numbers of women with low levels of education and/or from conservative rural settings, applying the MGI could become difficult. Use of the MGI may, for instance, require significant time and prompting from skilled counsellors to support women in expressing their postpartum experience [24]. At the same time, counsellors need to avoid influencing the women’s choices and expression.

An exploratory study that evaluated the use of the MGI in urban India found that all interviewed women identified only 2 to 5 areas of their life that had been affected by their pregnancy, after much suggestion and stimulation by the counsellors. The women in the study were perceived to have conceptual difficulty in identifying any areas or aspects of life which were positively affected by the birth [22]. The MAPP-QOL questionnaire, in turn, another specific instrument for postpartum QOL assessment, is self-administered and requires women to be able to read and write, but in our pilot study 45% of women had no or at most five years of schooling.

As we deemed the use of the MGI and MAPP-QOL instruments unfeasible for data collection on postpartum QOL within the MATIND project, we tested the use of the EQ-5D-5L questionnaire with rural Indian postpartum women in the states of Gujarat und Madhya Pradesh. Choosing the EQ-5D tool for postpartum QOL measurement was a pragmatic choice: Firstly, the EQ-5D instrument is available in many languages. Secondly, it can be administered in a number of modes, including face-to-face and telephone interviews. Thirdly, the EQ-5D questionnaire is relatively easy and quick to apply in comparison to the MGI or MAPP-QOL instruments. In addition, there had been some experience with using the EQ-5D questionnaire to evaluate postpartum QOL, yet mostly from high income countries [25–28].

### Appropriateness of using the EQ-5D an the Indian setting

The findings of the pilot study at hand indicate that the descriptive system of the EQ-5D-5L was able to depict and differentiate early postpartum QOL in Indian women by birth mode and over time. On average, at least 75% of the women in our study reported slight to extreme problems in four of the five health dimensions assessed by the EQ-5D-5L. The median woman who gave vaginal birth reported slight problems with mobility, self-care, usual activities, and pain or discomfort in the first interview 0–3 days after birth. At a similar postpartum time, the median woman who gave birth by cesarean section reported slight problems only with pain or discomfort and extreme problems with mobility, self-care and usual activities. No problems with anxiety or depression were reported by the median women in both birth groups, and less than 3% of the women interviewed ever reported more than slight problems with anxiety or depression. Among the women who gave vaginal births, the EQ-5D based QOL measure was lower for women who reported to have had an episiotomy during birth in comparison to those women who did not have this oftentimes discomforting procedure. Differences in the level of problems, and consequently QOL weights, between women with vaginal births and women with cesarean sections were highest at the time of the first interview which took place within the first three days postpartum. As time progressed, differences in QOL between women with vaginal and cesarean births narrowed and ceased to be statistically discernible at the time of the last follow-up interview when adjusting for socioeconomic, pregnancy and birth characteristics. Estimating the overall QOL in the first 21 days postpartum, vaginal births were associated with significantly more QALDs than cesarean sections.

The differences and trends in the postpartum QOL of Indian women, which were measured using the EQ-5D-5L in this pilot study, reflect plausible outcomes that are consistent with the findings of prior studies of postpartum QOL, conducted in other settings and/or using other QOL instruments [4–6]. Anxiety or depression was the EQ-5D dimension that was least affected by problems in our study. A recent systematic review and meta-analysis of postpartum depression in India, which included mostly studies that have used depression specific instruments, suggests a prevalence of postpartum depression of 22% (95% CI: 19%, 25%) overall and of 17% (95% CI: 14%, 21%) in rural areas [29]. In our study of the first postpartum month, 17% of women after vaginal birth and 26% after cesarean section reported slight to severe problems with anxiety or depression at the time of the first interview. By the end of our one-month pilot study, 97% of all women interviewed reported no problems with anxiety or depression. A decrease in the prevalence of possible depression within the first weeks postpartum is also suggested by the most comparable study, in terms of state and timing, of postpartum depression in the systematic review and meta-analysis. Using the Edinburgh Postnatal Depression Scale (EPDS), a prevalence of depression of 11%, 7% and 3% was found on postpartum days 1 and 6 and postpartum week 6, respectively, among randomly selected postpartum women in the Government Medical College in Gujarat [30], the state in which we recruited 74% of the women in our pilot study.

The fact that mostly slight if any problems with anxiety or depression were reported in our pilot study raises the question if the EQ-5D descriptive system is sensitive enough to detect changes in postpartum problems with anxiety or depression relevant to the QOL of Indian women. A systematic review of the EQ-5D responsiveness classified the overall responsiveness of the instrument to anxiety and depression disorders as small to large [31]. Future research might therefore need to study the existence and relevance of a possible lack of sensitivity in the anxiety or depression dimension when using the EQ-5D for postpartum QOL assessment in rural Indian women. Stigma expressing postpartum anxiety or stress, particularly when interviewed at home rather than in a hospital environment, could be a possible limitation to assessing problems with anxiety or depression in India [29]. Besides, difficulties to differentiate between postpartum blues and postpartum depression in the early postpartum period [29] might confound early postpartum period responses to questions about anxiety or depression. On the other hand, some researchers argue that postpartum stress in India can be low due strong extended family support during seminal moments in life like childbirth [32].

### Strengths and limitations

To our knowledge, this study was the first to assess postpartum QOL using the EQ-5D in a low resource setting. As a pilot study, this study has several limitations. Only part the EQ-5D questionnaire (the descriptive system) was assessed, and no validation whether the EQ-5D descriptive system measured postpartum QOL in Indian women accurately and consistently was performed. We can therefore not exclude that the descriptive system of the generic EQ-5D instrument failed to measure or inadequately measured health-related problems of Indian women in the postpartum period (see [33]). Further, due to the unavailability of an EQ-5D population norm, which would reflect the preferences over health states of the general public for India, a United Kingdom value set was used as an approximation to obtain QOL weights from the gathered problem levels. Finally, the pilot study’s sample, sample size and time horizon were pragmatic choices. Districts, health facilities and women were selected such that they could be reached well by research assistants. Applying a convenient sampling procedure resulted in a sample in which, for instance, the majority of women gave birth in a private health facility and all women with a cesarean section were primipara. While we adjusted for selected baseline differences in a multivariate analysis, there may be other confounders and lack of variation or statistical power in the data that influenced the results. The study was restricted to 4 weeks for logistical reasons even though the immediate postpartum period is commonly defined as up to 6 weeks after birth. However, 87% of all women in the study reported no problems in all five EQ-5D domains during the last interview. Therefore, impairments to QOL in the immediate postpartum period that can be measured with the EQ-5D-5L descriptive system appear to have been measured to a large extent in this pilot study. Changes in later postpartum QOL, beyond the immediate postpartum period, have not been studied.

## Conclusions

Postpartum QOL data from India is scarce, partly because of difficulties with implementing existing, specific postpartum QOL instruments in common Indian settings. We explored the feasibility of applying the generic EQ-5D-5L health questionnaire to postpartum women in India, of whom many had little or no formal education. We found that only the use of the descriptive part of the EQ-5D, which asks about the level of problems experienced in five generic areas of health, was feasible and acceptable to the rural women participating in our pilot study.

Subgroup analyses of problem levels, reported by women who gave birth in different ways, showed significant differences between the QOL of women who had a vaginal birth without episiotomy, those who had a vaginal birth with episiotomy and those who gave birth by cesarean section. As time progressed, postpartum QOL converged and ceased to be differed between groups by or before the end of the first postpartum month. Summing up the estimated QOL over the first 21 postpartum days, most QALDs were attained by women who gave vaginal birth without having an episiotomy. Significantly fewer QALDs were estimated for women who had an episiotomy during their vaginal birth, and again fewer for women who gave birth by cesarean section.

Differences and changes in the five health dimensions assessed by the EQ-5D-5L, as well as the estimated postpartum QOL and OALDs, reflect plausible health effects and recovery paths from vaginal and cesarean births. Finding results that are consistent with our expectations and many other studies of postpartum QOL suggests that measuring health-related QOL in the immediate postpartum period with the descriptive part of the generic EQ-5D-5L instrument can support assessments of maternal QOL in the postpartum period in India.

## Abbreviations

CS: cesarean section
CY: Chiranjeevi Yojana
EPDS: Using the Edinburgh Postnatal Depression Scale
EQ-5D-5L: EuroQoL-5 dimensions-5 levels
INR: Indian Rupee
IQR: interquartile range
JSY: Janani Suraksha Yojana
MAPP-QOL: Maternal Postpartum Quality of Life
MGI: Mother Generated Index
QALDs: quality-adjusted life days
QALYs: quality-adjusted life years
QOL: quality of life
VAS: visual analogue scale
VB: vaginal birth

## References

[1] Romano M, Cacciatore A, Giordano R, La Rosa B (2010). Postpartum period: three distinct but continuous phases. J Prenat Med.

[2] Brockington I (2004). Postpartum psychiatric disorders. Lancet.

[3] Firoz T, Chou D, von Dadelszen P, Agrawal P, Vanderkruik R, Tunçalp O (2013). Measuring maternal health: focus on maternal morbidity. Bull World Health Organ.

[4] Mogos MF, August EM, Salinas-Miranda AA, Sultan DH, Salihu HM (2013). A systematic review of quality of life measures in pregnant and postpartum mothers. Appl Res Qual Life.

[5] Rezaei N, Tavalaee Z, Sayehmiri K, Sharifi N, Daliri S (2018). The relationship between quality of life and methods of delivery: a systematic review and meta-analysis. Electron Physician.

[6] Rezaei S, Salimi Y, Zahirian Moghadam T, Mirzarahimi T, Mehrtak M, Zandian H (2018). Quality of life after vaginal and cesarean deliveries: a systematic review and meta-analysis. Int J Hum Rights Healthc.

[7] Symon A, Martin C (2012). The mother-generated index: a new approach to assessing maternal quality of life. Perinatal mental health: a clinical guide.

[8] Hill PD, Aldag JC, Hekel B, Riner G, Bloomfield P (2006). Maternal postpartum quality of life questionnaire. J Nurs Meas.

[9] Sidney K, de Costa A, Diwan V, Mavalankar DV, Smith H (2012). An evaluation of two large scale demand side financing programs for maternal health in India: the MATIND study protocol. BMC Public Health.

[10] Sidney K, Iyer V, Vora K, Mavalankar D, De Costa A (2016). Statewide program to promote institutional delivery in Gujarat, India: who participates and the degree of financial subsidy provided by the Chiranjeevi Yojana program. J Health Popul Nutr.

[11] Sidney K, Diwan V, El-Khatib Z, de Costa A. India’s JSY cash transfer program for maternal health: who participates and who doesn’t - a report from Ujjain district. Reprod Health 2012;9:2. 10.1186/1742-4755-9-2 PMC3287253 22269638

[12] van Reenen M, Oppe M. EQ-5D-5L user guide: basic information on how to use the EQ-5D-5L instrument. Rotterdam: EuroQol Research Foundation; 2015.

[13] Herdman M, Gudex C, Lloyd a, Janssen M, Kind P, Parkin D (2011). Development and preliminary testing of the new five-level version of EQ-5D (EQ-5D-5L). Qual Life Res.

[14] Janssen MF, Pickard a S, Golicki D, Gudex C, Niewada M, Scalone L (2013). Measurement properties of the EQ-5D-5L compared to the EQ-5D-3L across eight patient groups: a multi-country study. Qual Life Res.

[15] Szende A, Williams A, Measuring Self-Reported Population Health (2004). An international perspective based on EQ-5D.

[16] van Hout B, Janssen MF, Feng Y-S, Kohlmann T, Busschbach J, Golicki D (2012). Interim scoring for the EQ-5D-5L: mapping the EQ-5D-5L to EQ-5D-3L value sets. Value Heal.

[17] Matthews JN, Altman DG, Campbell MJ, Royston P (1990). Analysis of serial measurements in medical research. BMJ.

[18] Manca A, Hawkins N, Sculpher MJ (2005). Estimating mean QALYs in trial-based cost-effectiveness analysis: the importance of controlling for baseline utility. Health Econ.

[19] Gregory KD, Korst LM, Saeb S, Fridman M (2018). The role of patient-reported outcomes in women’s health. OBG Manag.

[20] Laine C, Davidoff F (1996). Patient-centered medicine. A professional evolution. JAMA.

[21] Bodhare TN, Sethi P, Bele SD, Gayatri D, Vivekanand A (2015). Postnatal quality of life, depressive symptoms, and social support among women in southern India. Women Health.

[22] Nagpal J, Dhar R, Sinha S, Bhargava V, Sachdeva A, Bhartia A (2008). An exploratory study to evaluate the utility of an adapted mother generated index (MGI) in assessment of postpartum quality of life in India. Health Qual Life Outcomes.

[23] Bele S. Postnatal quality of life: a neglected research area in India. Perspect Med Res. 2014;2:1–2.

[24] Vissandjée B, Abdool SN, Dupéré S (2002). Focus groups in rural Gujarat, India: a modified approach. Qual Health Res.

[25] Ride J, Lorgelly P, Tran T, Wynter K, Rowe H, Fisher J (2016). Preventing postnatal maternal mental health problems using a psychoeducational intervention: the cost-effectiveness of what were we thinking. BMJ Open.

[26] Petrou S, Kim SW, McParland P, Boyle EM (2017). Mode of delivery and long-term health-related quality-of-life outcomes: a prospective population-based study. Birth.

[27] Gerard Jansen AJ, Duvekot JJ, Hop WCJJ, Essink-Bot M-L, Beckers EAMM, Karsdorp VHMM (2007). New insights into fatigue and health-related quality of life after delivery. Acta Obstet Gynecol Scand.

[28] Shorten A, Shorten B (2012). The importance of mode of birth after previous cesarean: success, satisfaction, and postnatal health. J Midwifery Womens Health.

[29] Upadhyay RP, Chowdhury R, Salehi A, Sarkar K, Singh SK, Sinha B (2017). Postpartum depression in India: a systematic review and meta-analysis. Bull World Health Organ.

[30] Gokhale AV, Vaja A (2013). Screening for postpartum depression. Gujarat Med J.

[31] Payakachat N, Ali MM, Tilford JM (2015). Can the EQ-5D detect meaningful change? A systematic review. PharmacoEconomics.

[32] Wasan AD, Neufeld K, Jayaram G (2009). Practice patterns and treatment choices among psychiatrists in New Delhi, India. Soc Psychiatry Psychiatr Epidemiol.

[33] Chen T-H, Li L, Kochen MM (2005). A systematic review: how to choose appropriate health-related quality of life (HRQOL) measures in routine general practice?. J Zhejiang Univ Sci B.

